# The role of flaxseed and vitamin E on oxidative stress in prepubertal rats with experimental varicocele: An experimental study

**Published:** 2013-06

**Authors:** Shahla Sohrabipour, Adele Jafari, Mohamad Kamalinejad, Abdolfatah Sarrafnejd, Taherah Shahrestany, Hamid-Reza Sadeghipour

**Affiliations:** 1 *Molecular Medicine Research Center, Hormozgan University of Medical Sciences, Bandar Abbas, Iran.*; 2 *Department of Physiology, School of Medicine, Shahid Beheshti University of Medical Sciences, Tehran, Iran.*; 3 *Department of Pharmacogenosy, Faculty of Pharmacy, Shahid Beheshti University of Medical Sciences, Tehran, Iran.*; 4 *Department of Pathobiology, Faculty of Public Health, Tehran University of Medical Sciences, Tehran, Iran.*; 5 *Department of Physiology, School of Medicine, Tehran University of Medical Sciences, Tehran, Iran.*

**Keywords:** *Varicocele*, *Flax*, *Oxidative stress*, *Antioxidants*

## Abstract

**Background:** Prepubertal varicocele has the most devastating effects on the testes. Oxidative stress is the major cause leading to infertility in varicocele. The antioxidant properties of Flaxseed (FS) treatment in some oxidative diseases have been reported.

**Objective:** This study aimed to evaluate the antioxidant effect of FS in prepubertal rats with experimental varicocele.

**Materials and Methods:** Forty two male prepubertal rats were divided into 6 groups: the varicocele group were either fed with 10% FS, or with regular diet, or with Vit E, the group with sham operation fed with 10% FS, or had regular diet, and control rats who had not been operated but received regular diet. Varicocele was created by Koksal method. After 6 weeks sperm superoxide anion and H_2_O_2_ were evaluated by flowcytometery. Semen total antioxidant capacity (TAC) by Koracevic method and testes malondialdehyde (MDA) by thiobarbituric acid with spectrophotometry was measured.

**Results:** While superoxide anion and H_2_O_2_ were significantly higher in varicocele grop with regular diet (p=0.0001), FS significantly decreased the previously-mentioned parameters (p=0.0001). There were no significant differences for seminal TAC between 6 groups (p=0.07). Left testicular MDA concentration were lower in varicocele or group that were fed with 10% FS compared with other groups (p=0.001).

**Conclusion:** Reactive oxygen species (ROS) may cause sperm oxidative damage. FS as a fat soluble antioxidant can scavenge intracellular ROS production in varicocele.

## Introduction

The incidence of varicocele in Prepubertal age is up to 20%, which is similar to the incidence of varicoceles in adults. Varicocele in adolescence has the most devastating effects on the testes. The exact mechanism is not clear but oxidative stress is considered as a major cause that leads to infertility (1-8). 

The optimum age for treating a child with varicocele is still controversial (9). On the other hand just some cases of pediatric varicocele are infertile in the future and it remains difficult to identify which varicocele subjects should be treated (10-11). Surgical correction is not enough effective too (2, 9). So, most authors suggest a ‘‘wait and watch’’ approach to the evolution of pediatric varicocele (12). 

Therefore, it is still a worthwhile subject to investigate pharmacological treatment for varicocele related infertility. Antioxidant therapy is effective in reducing the oxidative stress in men or animals; although it is not clear which antioxidant and which dose is more effective in male infertility (13). Flaxseed (FS) is a nutritional whole grain, containing numerous chemical constituents and lignans that possess antioxidant activity (14). Lignan of matairesinol and secoisolariciresinol diglycoside (SDG) can be converted into mammalian lignans by colonic bacteria such as enterodiol and entrolactone that have antioxidant properties (14-15). In some studies the oxygen radical scavenging properties of FS lignans by inhibition of lipid peroxidation or direct hydroxyl radical scavenging activity are demonstrated (16). 

The efficiency of oral *FS* treatment in animal models of diabetes, endotoxic shock, lung injury and cancer has been noticed. One possible mechanism for FS is scavenging of radical species (17-19). In the proposed scheme, a radical abstracts a hydrogen atom from the lignan phenol hydroxyl, yielding a resonance-stabilized phenoxyl radical. The lignan radical could then react with another lignan radical, to form a stable product (20). Considering the evidence present in the current and older literature we have evaluated the antioxidant efficacy of FS in rats with prepubertal varicocele. Since FS is lipophilic antioxidant so it can easily pass the blood testis barrier and can induce direct antioxidant activity (14). It can also be orally assumed. Vitamin E is the most lipophilic antioxidant that is sometimes used in enhancing of sperm quality. 

Hence in our experiment FS antioxidant’s properties was only compared with vitamin E. This work aims to extend our original findings on the protective role of FS in scavenging ROS in prepubertal induced varicocele.

## Materials and methods

The study design was experimental intervention. Forty two male prepubertal Sprague-Dawley rats (25 days, 30-60 gr) were randomly divided in to 6 groups of seven rats. The first group was the control group and was fed with ordinary pellets. The second group underwent a sham operation. The third group (sham+FS) was sham operation that were fed base diet which was supplemented with 10% FS (wt/wt). 

The varicocele group (fourth group) underwent partial ligation of the left renal vein. The fifth group (varicocele+FS) consisted of varicocele rats that were fed base diet which was supplemented with 10% FS. In order to compare the antioxidant effects of FS10% with a standard fat soluble antioxidant we chose vitamin E (α tocopherol acetate, Sigma-Aldrich, Steinheim, Germany). Therefore, the sixth group (varicocele+vit E) consisted of the varicocele group by injection of vitamin E (IM-100 mg kg^-1^/3 days in a week) (21). FS diet was the basal diet by adding 10% (wt/wt) freshly whole ground FS. The diets were stored at 4^o^C to avoid oxidative degradation and fresh diet was provided every 3 days (17-19, 22-24). To induce the varicocele to the animals, the rats were firstly anaesthetized with intra-peritoneal injection xylazine (15 mg/kg^-1^) and ketamine (75 mg/kg^-1^). 

As a method described by Koksal *et al*, the upper left abdominal quadrant was approached through a midline laparotomy incision (25). The abdominal contents were gently pushed to the right. A tunnel was made under the left renal vein proximal to the venacava with tearing. By using the 24-gauge angiocatheter the left renal vein was ligated up to 50% with silk 4/0. An angiocatheter was interposed at the point medial to the insertion of the adrenal and spermatic vein into the left renal vein. The ligature was placed around the renal vein over the parallel angiocatheter. After placing the ligature, the angiocatheter was removed and abdominal wall was repaired (25). 

Six weeks later, the left spermatic vein was checked for significant dilation and renal atrophy. Dilation of the internal spermatic veins was identified during this procedure as an internal control. All rats in experimental group which failed dilation or presence renal atrophy were excluded from the study (n=5). Sham operated prepubertal rats underwent a similar procedure except for the ligation. Samplings were conducted after 6 weeks when all rats were adults. After laparatomy the left and right caudal epididymis of the rats was used in sperm collection. 

The caudal epididymis was placed into a Petri dish containing 1 mL of normal saline at 37^o^C for 5 minutes. For flow cytometric assays, sperm cells were separated by centrifugation (500g, 3 minutes, 37^o^C) to remove pellet serum. The cells were then resuspended in phosphate-buffered saline (PBS; pH=7.4, 37^o^C) to a final concentration of 1×10^6^ cells/mL^-1^. To detect intracellular superoxide anion production, parallel samples were stained with Dihydroethidium (DHE) using 2 protocols, first DHE procedure, aliquots of 1×10^6^ cells were incubated in PBS containing 100 μmol DHE (Sigma-Aldrich, Steinheim, Germany) for 15 minutes at 37^o^C in a dark place; then the samples were washed with PBS and immediately analyzed. 

In the second DHE procedure, cells were stained with DHE, as described above. After being washed with PBS, the samples were loaded with menadione (1 mmol; Axxora Deutschland, Germany), incubated for 1 hour, and then analyzed immediately. Menadione can induce superoxide anion production and was used for the positive control group (26). To detect H_2_O_2_ in sperms, aliquots of 1×10^6^ cells were incubated in PBS containing 10 μmol 2′, 7′-Dichlorofluorescin diacetate (DCF) (Sigma- Aldrich, Steinheim, Germany) for 30 minutes at room temperature in a dark place; in the second DCF procedure, cells were stained with DCF, as described above. 

After being washed with PBS, the samples were loaded with 5μl H_2_O_2_ as positive control, incubated for 20 min. The samples were then analyzed immediately (27). Before analysis, all aliquots were counterstained with propidium iodide (PI) (Sigma- Aldrich, Steinheim, Germany) which assesses cell viability. This fluorochrome can enter dead cells through their damaged plasma membrane. The samples stained with DCF and DHE were then incubated in PI with 23.9 μmol for 5 minutes at 37^o^C in the dark and immediately analyzed (26).

All samples were analyzed with a Partec PAS FacScan Flow Cytometer (DAKO Cytomation, Denmark) with a 488-nm excitation laser and Flowmax software. A forward and side scatter gate was used to select single sperms out of clumps and debris. Approximately 10,000 gated events were analyzed per sample. Fluorescence from the DHE- stained sperm was collected in a fluorescence detector FL2 with a 585-nm and and DCF in a FL1 530 nm bandpass filter. To limit the evaluation of DHE fluorescence to viable spermatozoa, only the subpopulation of PI-negative sperm was included in the evaluation. 

Fluorescent measurements were compensated to minimize spillover fluorescence between red and green spectrum. Data were reported as fluorescence intensity. Seminal plasma total antioxidant activity (Total Antioxidant Capacity, TAC) was measured using the Koracevic method. The assay measured the capacity of seminal plasma to inhibit the production of thiobarbituric acid reactive substance (TBARS) from sodium benzoate under the influence of ROS generated through the Fenton reaction. Uric acid solution was used as the standard (28). Malondialdehyde (MDA) is a stable product of lipid peroxidation. MDA assay was performed with thiobarbituric acid test in the supernatant, according to the method suggested by Cheeseman and Esterbaue (29). 

The testes MDA results are expressed as nmol gr tissue^-1^. MDA reacts with thiobarbituric acid to give a red compound absorbing at 532 nm in a spectrophotometer. After laparatomy both testes were delivered into the abdomen. Testis tissue was excised from fat and adhering tissues and washed in cold saline, then transferred to a -70^o^C freezer. Tissues were homogenized in phosphate buffer (pH=7.4) and the supernatant was used for MDA experiments. The protocol of this study was approved by the Ethics Committee of Hormozgan University of Medical Sciences )HEC-91-4-6(.

**Statistical analysis**

The statistical analysis was performed with SPSS for windows (SPSS, Chicago, II). Numerical data were expressed as mean±SE. Statistical analysis was performed using one-way analysis of variance among groups and t test for comparing right and left data. The Kruskal-Wallis test was used when the distribution of data was not normal. P-value ≤0.05 was considered as statistically significant.

## Results

**Figure 1:**
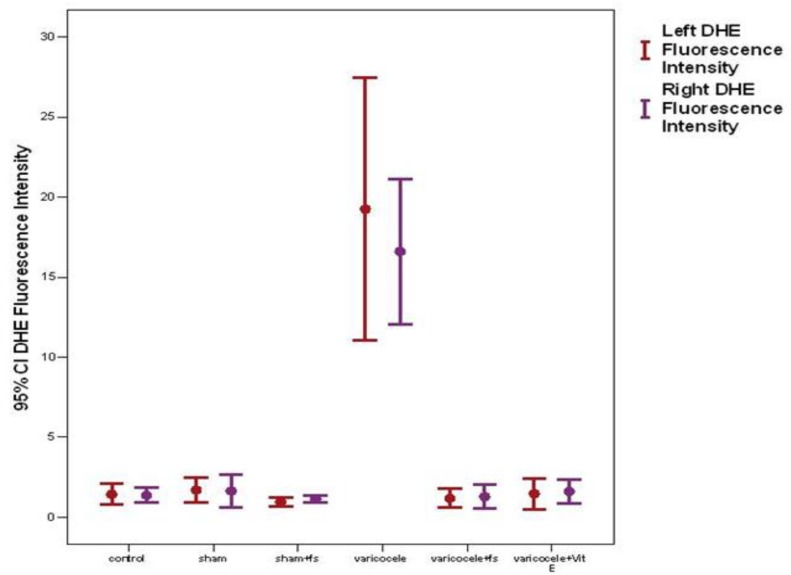
The effect of FS 10% on superoxide anion production in sperm cells on the left and right side

**Figure 2:**
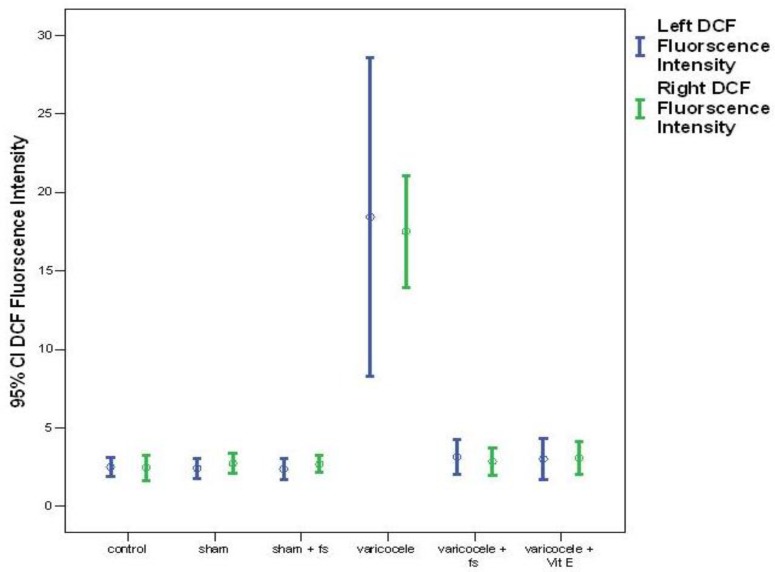
The effect of FS 10% on H_2_O_2_ production on the sperm cells in left and right sides

Dietary FS supplementation ameliorates sperm injury after varicocele. Intracellular superoxide anion production, as evaluated by using the fluorescence intensity of DHE-positive sperm cells, was significantly higher in varicocele groups compared with the control and sham groups both on the left and right sides (p=0.0001). But in varicocele+FS and varicocele+Vit E group it decreased significantly (p=0.0001). There was no difference between varicocele+FS and varicocele+Vit E groups (p=0.08, Figure 1). H_2_O_2_ production, as evaluated using the fluorescence intensity of DCF-positive sperm cells, was significantly higher in varicocele group compared with the control and sham groups both on the left and right sides (p=0.0001). But it decreased significantly in varicocele+FS and varicocele+Vit E groups. (p=0.0001). There was no difference between varicocele+FS and varicocele+Vit E groups (p=0.08, Figure 2). 

The effect of flaxseed on total antioxidant capacity in the cudal epididymis in varicocele is shown in table I. There was no significant difference in the seminal plasma total antioxidant capacity among all groups (p=0.07). Testicular MDA concentration of varicocele+FS rats was lower on the left side (10.42±1.63) compared with varicocele groups (13.93±1.04), p=0.001). 

Consuming FS as Vitamin E (9.90±2.40, p=0.001) decreased testicular MDA. No significant difference was seen between varicocele+FS and varicocele+vitamin E (p=0.06, Table I).

**Table I:** Effect of *FS* 10% on testicular MDA and cudal epididim TAC on the left and right side

	**MDA (nmol gr tissue** ^-1^ **)**			**TAC (mmol Lit** ^-1^ **)**		
	**Left side**	**Right side**	**Left side**		**Right side**
Control	8.96 ± 1.1	9.26 ± 0.81	0.29± 0.02		0.27 ± 0.02
Sham	8.86 ±0.9	7.88 ± 0.76	0.25 ± 0.02		0.23 ± 0.01
Sham+ *FS*	9.1 ± 0.96	8.07 ± 0.7	0.28 ± 0.01		0.26 ± 0.02
Varicocele	13.93 ± 0.39*	10.21 ± 0.49	0.23 ± 0.01		0.2 ± 0.03
Varicocele+ *FS*	10.42 ± 0.61^≠^	8.88 ± 0.58	0.22 ± 0.02		0.16 ± 0.02
Varicocele+ vitamin E	9.9 ± 0.9^≠^	7.6 ± 0.59	0.16 ± 0.01		0.21 ± 0.01

MDA= Malondialdehyde.

TAC= Total Antioxidant Capacity.

Values are mean±SEM

* Signiﬁcant difference compared to control and sham groups (p<0.05).

≠ Significant difference compared to varicocele group (p≤0.05).

## Discussion

Our results showed a significant increase in the production of superoxide anion and H_2_O_2 _inside the sperms in both sides in rat with varicocele compared with the control and sham groups. However, rats supplemented with FS had a lower production level of superoxide anion and H_2_O_2_. So it seems that dietary FS are protective against oxidative stress in rats with varicocele. There was no similar study to ours but the oxygen hydroxyl radical scavenging activity of FS has been shown in previous studies (18, 24). 

FS showed antioxidant activities in diabetes, in carbon tetrachloride-induced oxidative stress, radiotrapy, cancer and also in invitro models (17-19, 23, 30, 31). Some antioxidants like fat soluble vitamin E neutralize radical species by forming stable non- radical products. It is possible that FS and vitamin E have similar mechanisms. A possible mechanism for lignans of FS is described by Hosseinian *et al.* In this proposed mechanism a radical can abstract a hydrogen atom from the lignan phenol hydroxyl and produce a stable radical so that the lignan radical reacts with a second radical and forms a stable product (20).

Salama *et al* reported that polymorph nuclear leukocytes accumulate in testicular tissue of rats with varicocele (32). There are some reports about the ability of FS in inhibition of oxygen free radicals by neutrophils, so the other possible benefit of FS in varicocele is that FS lignans can inhibit the production of ROS by neutrophils (14, 24).

Although, the TAC level of seminal plasma among the groups was not statistically significant, as reported by Elsayed *et al*, the possible clarification is mobilization of antioxidant from other organs, for example the liver response to oxidative stress (33). Jafari *et al* also reported no change in TAC level in adult varicocele induction rats (26). In some clinical studies there was also no difference between TAC in seminal plasma of the control group and patients with varicocele (34). 

In our finding, FS has no effect on TAC. Although in Naqshbandi *et al* research dietary supplementation of flaxseed oil in Cisplatin treated rats increased the activities of catalase, superoxide dismutase and glutathione peroxidase (35). MDA level measurements are widely used as an indicator of lipid peroxidation. In some adolescent patients with varicocele, the MDA level increases (36). 

In our study, left testes tissue levels of MDA increased in rats with varicocele compared with the control and sham groups. Rats fed with FS 10% or those that received vitamin E had a significantly lower level of MDA on the left side. There was no similar study to compare but other studies confirm the effect of FS in decreasing MDA levels in other tissues. 

Lee *et al* showed that mice that were fed FS 10% and then underwent pulmonary ischemia reperfusion had a lower level of MDA in the pulmonary tissue (16). In Kinniry *et al *study, the same effect was also seen in the lung tissue. In rats with diabetes mellitus, SDG (the lignan of FS) treatment lowered serum and pancreatic MDA, and diabetes did not develop. FS lignan like SDG has antioxidant activity and they can scavenge of reactive oxygen specious (ROS) production (17). Solomidou *et al* reported that, mice fed with a 10%FS diet (whether given preventively or therapeutically) maintained lower MDA level at 4 months post- X-ray radiation therapy (23).

In a randomized crossover research in obese and diabetic patients’ consumption of FS in form of ground grain or bread for 12 weeks decreased TBARS. TBARS is an indicator for lipid peroxidation, and it is measured in MDA equivalents. FS decreases lipid peroxidation by scavenging hydroxyl radical (23). Increased production of ROS and/or the decrease of local antioxidant capacity, result in oxidative stress impairment of sperm parameters in varicocele. Several studies have reported the effects of increased oxidative stress in the serum, semen, and testicular tissues of patients with varicocele. 

However, the etiology of oxidative stress elevation in association with varicoceles is unclear (30). A metaanalysis of four studies published in 2006 showed patients with varicocele had significantly greater ROS and lower TAC levels. Similar to the findings in the spermatic vein, the finding of increased MDA and NO levels in the semen of patients with varicocele is also suggestive of oxidative stress (36). Several naturally occurring antioxidants have been studied in stress oxidative related disease, but some may produce undesirable side effects, thus limiting their benefit under which it can be used safely (24). Flaxseed is a safe, economically affordable nutritional agent, which is currently used in a number of clinical trials and can thus prove to be a useful alternative (30, 37). 

Dietary flaxseed has high contents of omega-3 fatty acid and lignans with antioxidant properties (16). Some study show that plasma levels of FS lignans in human subjects that fed 25 g of raw FS for 8 days is comparable to rodent. If the bioactive components of FS can be isolated and formulated, it would be effective in stress oxidative related disease (24). One of the limitations of our study is that pregnancy rate was not measured. Pregnancy rate is the most effective outcome in varicocele treatment. Unfortunately we did not measure DNA fragmentation. FS could reduce DNA fragmentation too. We conclude that dietary FS can reduce sperm and testis oxidative damage in prepubertal varicocele model and this protective effect may in part be mediated by scavenging of ROS. However there is a need for prospective randomized clinical trials including pregnancy rates on the basis of this experimental observation to reach definitive conclusion. 

## Conflict of interest

The authors declare that there is no conflict of interest in this article.
